# A Role for Protein Phosphatase 2A in Regulating p38 Mitogen Activated Protein Kinase Activation and Tumor Necrosis Factor-Alpha Expression during Influenza Virus Infection

**DOI:** 10.3390/ijms14047327

**Published:** 2013-04-02

**Authors:** Anna H. Y. Law, Alex H. M. Tam, Davy C. W. Lee, Allan S. Y. Lau

**Affiliations:** Cytokine Biology Group, Department of Paediatrics and Adolescent Medicine, Li Ka Shing Faculty of Medicine, the University of Hong Kong, Pokfulam, Hong Kong, China; E-Mails: lawanna@hku.hk (A.H.Y.L.); tempo927@yahoo.com.hk (A.H.M.T.); dcwlee@hku.hk (D.C.W.L.)

**Keywords:** protein phosphatase 2A, tumor necrosis factor-alpha, p38 mitogen activated protein kinase, influenza virus

## Abstract

Influenza viruses of avian origin continue to pose pandemic threats to human health. Some of the H5N1 and H9N2 virus subtypes induce markedly elevated cytokine levels when compared with the seasonal H1N1 virus. We previously showed that H5N1/97 hyperinduces tumor necrosis factor (TNF)-alpha through p38 mitogen activated protein kinase (MAPK). However, the detailed mechanisms of p38MAPK activation and TNF-alpha hyperinduction following influenza virus infections are not known. Negative feedback regulations of cytokine expression play important roles in avoiding overwhelming production of proinflammatory cytokines. Here we hypothesize that protein phosphatases are involved in the regulation of cytokine expressions during influenza virus infection. We investigated the roles of protein phosphatases including MAPK phosphatase-1 (MKP-1) and protein phosphatase type 2A (PP2A) in modulating p38MAPK activation and downstream TNF-alpha expressions in primary human monocyte-derived macrophages (PBMac) infected with H9N2/G1 or H1N1 influenza virus. We demonstrate that H9N2/G1 virus activated p38MAPK and hyperinduced TNF-alpha production in PBMac when compared with H1N1 virus. H9N2/G1 induced PP2A activity in PBMac and, with the treatment of a PP2A inhibitor, p38MAPK phosphorylation and TNF-alpha production were further increased in the virus-infected macrophages. However, H9N2/G1 did not induce the expression of PP2A indicating that the activation of PP2A is not mediated by p38MAPK in virus-infected PBMac. On the other hand, PP2A may not be the targets of H9N2/G1 in the upstream of p38MAPK signaling pathways since H1N1 also induced PP2A activation in primary macrophages. Our results may provide new insights into the control of cytokine dysregulation.

## 1. Introduction

Influenza viruses continue to pose pandemic and epidemic threats to the public. Infection of humans with the highly pathogenic avian influenza H5N1 virus is associated with mortality in excess of 60% [1]. Fatal outcomes of H5N1 virus infections are associated with high viral load and hypercytokinemia [2]. Avian H9N2 Quail/HK/G1/97 (H9N2/G1) virus is one of the precursor viruses of the highly pathogenic H5N1/97 virus [3]. With the prevalent circulations of H9N2 virus in quail in southern China [4] and several zoonotic transmission cases [5–8], H9N2 virus may evolve into a human-adapted avian influenza virus with pandemic potential [9]. We have previously demonstrated that H5N1/97 virus hyperinduces tumor necrosis factor (TNF)-alpha expression in primary monocyte-derived macrophages (PBMac), which is mediated by p38 mitogen activated protein kinase (MAPK) [10]. Recent reports, including ours, showed that H9N2/G1 virus also hyperinduces proinflammatory cytokines and chemokines comparable to H5N1/97 [11,12]. However, the roles of p38MAPK and other MAPK family members in H9N2/G1-induced TNF-alpha expression in PBMac are still unclear.

MAPK signaling pathways play key roles in the induction of inflammatory reactions including production of proinflammatory cytokines such as TNF-alpha, IL-1β and IL-6. Since the uncontrolled cytokine productions can lead to detrimental effects to the host, activation of MAPK also induces the negative feedback mechanisms to regulate the immune responses. Hence, MAPK and phosphatases are key players in the regulation of pro- and anti-inflammatory responses in the host. Reports have shown that MAPK phosphatase-1 (MKP-1) and protein phosphatase type 2A (PP2A) are two well-known negative regulators of p38MAPK. MKP-1 deficient mice are susceptible to septic shock [13,14]. In response to lipopolysaccharide, macrophages from MKP-1 deficient mice show prolong activation of p38MAPK and enhanced production of TNF-alpha and IL-6 [15]. PP2A constitutes a large family of Ser/Thr phosphatases, which are involved in diverse cellular functions [16]. PP2A suppresses the activations of p38MAPK and the upstream kinases of MAPK signaling pathways [17–19]. It has been shown that viruses develop various strategies to target PP2A in order to counteract host antiviral responses [20]. However, the roles of MKP-1 and PP2A in cytokine regulation during influenza virus infections have not been reported. Here, we show that H9N2/G1 virus activated p38MAPK and hyperinduced production of TNF-alpha in PBMac when compared with H1N1 virus. Moreover, the phosphorylation levels of p38MAPK and subsequent TNF-alpha expression in H9N2/G1-infected macrophages were further increased by the treatment of a selective inhibitor of PP2A, okadaic acid (OA). Interestingly, OA also increased the levels of p38MAPK phosphorylation and TNF-alpha mRNA in H1N1-infected macrophages. These results provide insights into the complex mechanisms of MAPK-mediated cytokine production during influenza virus infections.

## 2. Results and Discussion

### 2.1. H9N2/G1 Hyperinduced TNF-alpha Expression through the Activation of p38MAPK in Primary Human Blood Macrophages

**Figure 1:**
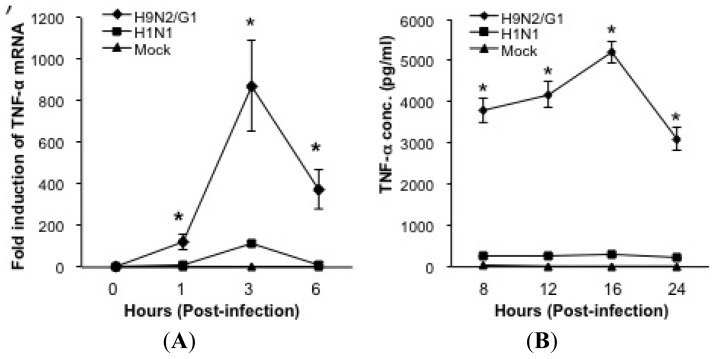

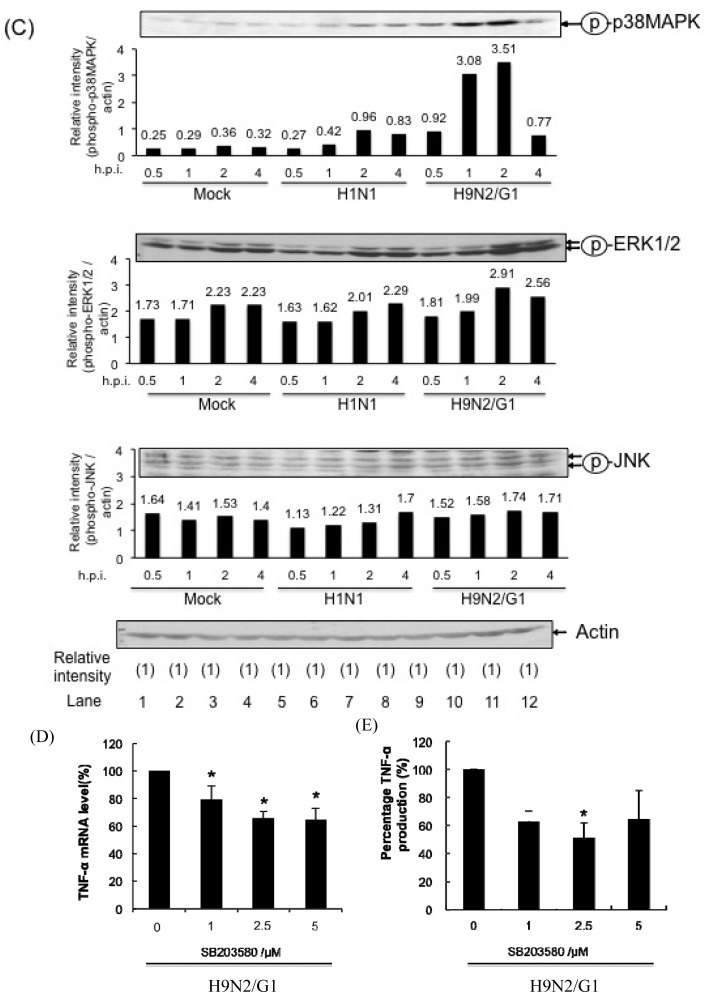
H9N2/G1 hyperinduces tumor necrosis factor (TNF)-alpha expression *via* the activation of p38MAPK in primary human blood macrophages. Primary human monocyte-derived macrophages (PBMac) were mock-treated or infected with H9N2/G1 or H1N1 at a multiplicity of infection (m.o.i.) of 2. Total RNA, culture supernatant or cell lysates were collected at the indicated time points. (**A**) Quantitative RT-PCR analysis of TNF-alpha mRNA by using TaqMan Gene Expression Assay in H9N2/G1 or H1N1-infected PBMac; (**B**) TNF-alpha levels in cell culture supernatant of the H9N2/G1 or H1N1-infected PBMac as determined by ELISA; (**C**) Western blot analysis of the phosphorylation of MAPKs. The phosphorylation levels of p38MAPK, ERK1/2 and JNK were determined by using specific antibodies. Actin was used as a loading control. Protein band intensities were determined by using Bio-Rad Quantity One imaging software. The band intensities of the phosphorylated proteins were normalized to the corresponding actin intensities. The normalized values are presented in the graphs. Representative figure of experiments from five independent blood donors is shown; (**D** & **E**) PBMac were pre-treated with the indicated doses of p38MAPK inhibitor (SB203580) for 30 min prior to H9N2/G1 infections; (**D**) The levels of TNF-alpha mRNA at 3 h.p.i. were examined by TaqMan Gene Expression Assay; (**E**) TNF-alpha protein in culture supernatants at 16 h.p.i. were measured by ELISA. The relative TNF-alpha mRNA and protein levels in the SB203580-treated cells compared to the mock-treated cells (DMSO) are shown. *p < 0.05; h.p.i., hour post infection; ELISA, enzyme-linked immunosorbent assay; DMSO, dimethylsulphoxide.

Our previous reports demonstrate that avian influenza viruses including H5N1 and H9N2/G1 induce high levels of TNF-alpha production in macrophages in contrast to those cells infected with human influenza virus subtypes H3N2 and H1N1 [10,11]. In addition, H5N1-induced TNF-alpha expression is, in large part, mediated by the activation of p38MAPK [10]. To investigate the mechanisms of p38MAPK-mediated hyperinduction of TNF-alpha, we examined the kinetics of p38MAPK activation and TNF-alpha expression in PBMac infected with H9N2/G1 or H1N1 virus. PBMac were mock-treated or infected with H9N2/G1 or H1N1 at a multiplicity of infection (m.o.i.) of 2. At indicated time points, total RNA of treated cells were harvested for examining the levels of TNF-alpha mRNA by using TaqMan Gene Expression Assay as previously reported [10]. The levels of TNF-alpha production in culture supernatants were measured by using enzyme-linked immunosorbent assay (ELISA). Like H5N1 viruses, H9N2/G1 significantly increased TNF-alpha levels when compared with H1N1-infected PBMac (Figure 1A,B). We then examined the phosphorylation levels of p38MAPK, ERK1/2, and JNK in the H9N2/G1- or H1N1-infected PBMac by using Western blot analysis. H9N2/G1 activated p38MAPK and ERK1/2, but not JNK, from 1 to 2 h post-infection (h.p.i.) (Figure 1C, lanes 9–12) when compared with the mock-treated control (Figure 1C, lanes 1–4) or H1N1-infected PBMac (Figure 1C, lanes 5–8). Moreover, the levels of p38MAPK phosphorylations induced by H9N2/G1 were higher than those of ERK1/2 (Figure 1C, lane 11).

Furthermore, we examined the roles of p38MAPK in the TNF-alpha induction by using a specific p38MAPK inhibitor, SB203580. PBMac were treated with SB203580 at the indicated doses for 30 min, and then infected with H9N2/G1 virus. The levels of TNF-alpha mRNA and protein were measured at 3 h.p.i. and 16 h.p.i., respectively. Notably, the levels of TNF-alpha mRNA and protein in H9N2/G1-infected PBMac were significantly suppressed by SB203580 and in a dose dependent manner (Figure 1D,E). The suppressive effect of SB203580 on the activities of p38MAPK was shown in supplementary figure (Figure S1A) and SB203580 did not show cytotoxic effects on the H9N2/G1-infected PBMac (Figure S1B). By examining the levels of influenza virus nucleoprotein using Western blot, we show that SB203580 did not affect the expression level of the viral protein suggesting that SB203580 did not interfere with H9N2/G1 infection (Figure S1C). To summarize, H9N2/G1 induced a significantly higher level of TNF-alpha when compared with the seasonal H1N1 and the hyperinduction was mediated through p38MAPK.

### 2.2. H9N2/G1 Virus Did Not Alter the Cellular Protein Levels of MKP-1 and PP2A Catalytic Subunit

**Figure 2:**
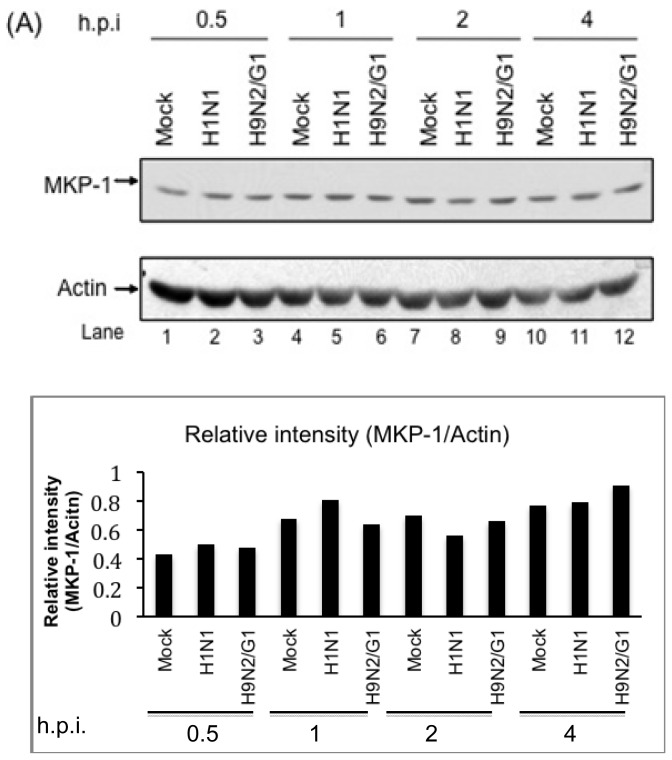

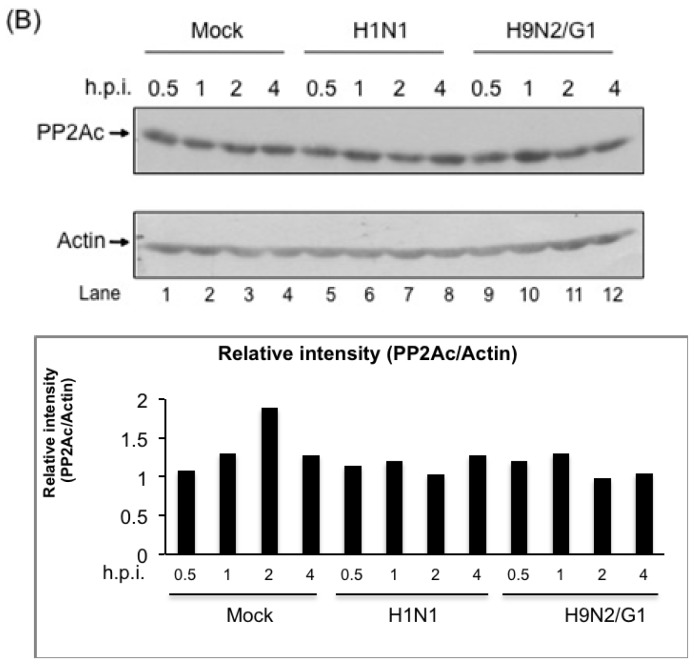
Expression levels of MKP-1 and PP2A catalytic subunit were not affected by H9N2/G1 and H1N1 infections. Primary human monocyte-derived macrophages (PBMac) were mock-treated, and infected with H1N1 or H9N2/G1 viruses. Total cell lysates were harvested at the indicated time points after infections and the expression levels of MKP-1 (**A**) and PP2A catalytic subunit (PP2Ac) (**B**) were examined by Western blot analysis. Representative figure of experiments from five independent blood donors is shown. Actin was used as a loading control. Graphical presentation of the expressions of MKP-1 and PP2A were shown in the lower panel of (**A**) and (**B**). The densities of the protein bands were determined by using Bio-Rad Quantity One imaging software. The MKP-1 (**A**) and PP2Ac (**B**) levels were normalized with the corresponding actin levels. The values in the graphs represent the normalized values. h.p.i., hour post infection.

To investigate the involvement of the negative regulators of p38MAPK upon influenza virus infections, we measured the expressions of MKP-1 and PP2A catalytic subunit in H9N2/G1- or H1N1-infected PBMac at the indicated time points. We show that H9N2/G1 and H1N1 did not induce the expression of MKP-1 from 0.5 to 4 h.p.i. when compared with the mock-treated cells (Figure 2A).

Previous reports have shown that HIV or coronavirus induces MKP-1 expression which in turn to modulate the excessive production of cytokines in virus-infected cells [21,22]. However, our results show that MKP-1 expression was not enhanced by the influenza viruses. Similar to MKP-1, PP2A expressions were not modulated by H9N2/G1- or H1N1-infected PBMac (Figure 2B). These results suggest that the regulation of MKP-1 and PP2A expressions are dispensable to the p38MAPK activity in influenza virus-infected PBMac.

### 2.3. H9N2/G1 Induced PP2A Activity in Primary Human Blood Macrophages

**Figure 3:**
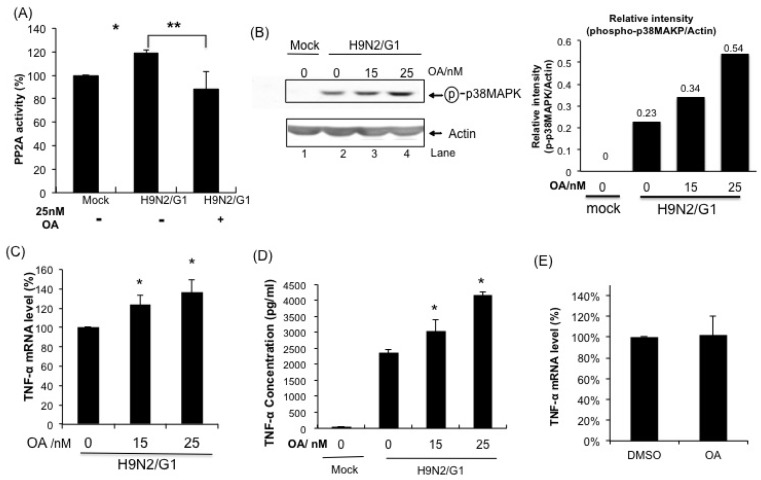
Involvement of PP2A in modulating the activation of p38MAPK phosphorylation and TNF-alpha hyperinduction in H9N2/G1-infected primary human blood macrophages. (**A**) Primary human monocyte-derived macrophages (PBMac) were mock-treated or treated with 25 nM okadaic acid (OA) for 30 min prior to H9N2/G1 virus infection. At 2 h.p.i., the PP2A activity was quantified by using the PP2A Phosphatase Assay Kit (Millipore); (**B**) **Left panel**: Effects of OA on the p38MAPK phosphorylation. PBMac were treated with the indicated doses of OA for 30 min prior to H9N2/G1 infection. At 2 h.p.i., cell lysates were collected and the phosphorylation levels of p38MAPK were determined by using Western blot analysis. Representative figure of experiments from five independent blood donors is shown; **Right panel**: graphical presentation of the Western blot analysis. The densities of the protein bands were determined by using Bio-Rad Quantity One imaging software. Actin was used as a loading control. The band intensities of phosphorylated p38MAPK were normalized with those of actin. The numbers shown above the columns represent the normalized values; (**C**,**D**) Macrophages were treated with the indicated doses of OA for 30 min prior to H9N2/G1 infection. The TNF-alpha mRNA levels (**C**) and protein levels in culture supernatant (**D**) were measured by using quantitative RT-PCR and ELISA, respectively; (**E**) PBMac were treated with DMSO or 25 nM OA for 3 h. The total RNA samples were then harvested and the TNF-alpha levels were quantified by using quantitative RT-PCR. Experiments were performed by using blood from at least five independent donors. *p < 0.05; **p < 0.01. h.p.i., hour post infection; ELISA, enzyme-linked immunosorbent assay; DMSO, dimethylsulphoxide.

Whether the activity of PP2A was modulated during H9N2/G1 infection, we examined the PP2A activity in the H9N2/G1-infected cells by using the PP2A Phosphatase Assay Kit (Millipore, Billerica, MA, USA) (Figure 3A). Surprisingly, despite H9N2/G1 induced high level of p38MAPK phosphorylation, the PP2A activity was increased when compared with the mock-treated cells. The activity of PP2A induced by H9N2/G1 was abrogated by okadaic acid (OA), a selective PP2A inhibitor, at 25 nM (Figure 3A). OA is a reversible, non-competitive inhibitor of the serine/theorine protein phosphatases PP1 and PP2A [23]. However, OA inhibits PP2A activities at 1–2 nM whereas it inhibits PP1 above 1 μM in tissue extracts [24]. Meanwhile, cytotoxicity of OA in PBMac was examined (Figure S2). PBMac were treated with OA at the indicated concentrations and the survivals of treated cells were measured by MTT assay after incubating for 4 to 24 h. Results show that OA did not induce cell death in PBMac.

### 2.4. PP2A Is a Negative Regulator in H9N2/G1-Induced p38MAPK Activation and TNF-Alpha Expression

To investigate the roles of virus-activated PP2A in the regulation of p38MAPK upon H9N2/G1 infection, we pre-treated the PBMac with OA and examined the p38MAPK phosphorylation levels in the H9N2/G1-infected PBMac at 2 h.p.i. by using Western blot analysis. The phosphorylation levels of p38MAPK in H9N2/G1-infected PBMac were enhanced by the OA treatment in a dose-dependent manner (Figure 3B, lanes 3 and 4). In contrast, p38MAPK was not activated in PBMac with OA treatment only (Figure S3). These results show that PP2A was involved in suppressing the p38MAPK activation in H9N2/G1-infected cells.

We then examined the effects of OA on H9N2/G1-induced TNF-alpha production [10]. As shown in Figure 3C,D, OA further increased the levels of TNF-alpha mRNA and protein in H9N2/G1-infected PBMac and in a dose-dependent manner. However, OA did not induce the TNF-alpha mRNA level in uninfected PBMac (Figure 3E). Our results revealed that PP2A played a role in damping the p38MAPK-mediated TNF-alpha production in PBMac with H9N2/G1 infection.

### 2.5. H1N1 also Induces PP2A Activation in Primary Human Blood Macrophages

**Figure 4:**
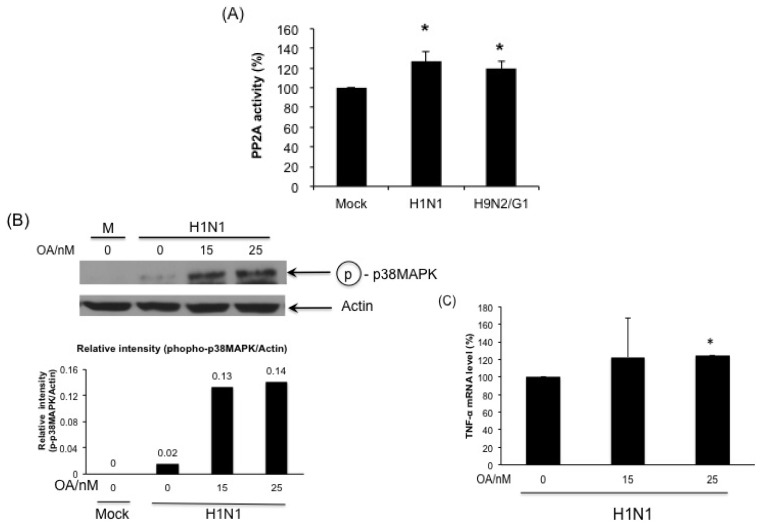
Involvement of PP2A in modulating the activation of p38MAPK phosphorylation and the TNF-alpha hyperinduction in H1N1-infected primary human blood macrophages. (**A**) Primary human monocyte-derived macrophages (PBMac) were mock-treated, infected with H1N1 or H9N2/G1 at a m.o.i. of 2. At 2 h.p.i., total cell lysates were harvested and PP2A activity was quantified by using PP2A Phosphatase Assay Kit (Millipore, Billerica, MA, USA); (**B**) PBMac were mock-treated or treated with 15 or 25 nM okadaic acid (OA) for 30 min prior to H1N1 infection. **Upper panel**: total protein lysates were harvested at 2 h.p.i. and phosphorylated p38MAPK was detected by using Western blot analysis. Actin was used as a loading control. **Lower panel**: the band intensities in the upper panel were measured by Quantity One imaging software (Bio-Rad, Hercules, CA, USA). The relative band intensities of the phosphorylated p38MAPK compared to actin were presented; (**C**) Total RNA from H1N1-infected cell treated with OA were harvested at 3 h.p.i.; TNF-alpha mRNA levels were measured by TaqMan Gene Expression Assay. Experiments were performed by using blood from at least three independent donors. *p < 0.05.

Whether PP2A activation is specific to H9N2/G1 infection, we measured the PP2A activity in H1N1-and H9N2/G1-infected PBMac at 2 h.p.i. Our results show that H1N1 also induced PP2A activity and there was no significant difference of PP2A activity between H1N1- and H9N2/G1-infected cells (Figure 4A). In addition, blocking the PP2A activity by OA increased the phosphorylation of p38MAPK and TNF-alpha mRNA in H1N1-infected PBMac (Figure 4B,C) suggesting that PP2A activation is not specific to H9N2/G1 infection.

Signaling cascades of MAPK and phosphatases play critical roles in the regulation of proinflammatory cytokines production during microbial infections to prevent the uncontrolled inflammatory reactions that lead to the deleterious effects on the host. We previously reported p38MAPK mediates, in part, the hyperinduction of TNF-alpha in primary human blood macrophages with H5N1/97 infection [10]. In the current study, we further investigate the negative regulatory mechanisms of p38MAPK-mediated hyperinduction of TNF-alpha in influenza virus infections. Our results show that H9N2/G1, like H5N1/97 virus, induced p38MAPK activation and high level of TNF-alpha production in macrophages when compared with the seasonal H1N1 virus. Moreover, H9N2/G1 induced the activation of PP2A but not MKP-1in the infected cells. With the treatment of PP2A inhibitor, the levels of p38MAPK activity and TNF-alpha production were enhanced suggesting PP2A plays a role in negative regulation of p38MAPK activation in H9N2/G1 infection. However, H9N2/G1 did not induce PP2A expression indicating that the activation of PP2A is not mediated by p38MAPK. On the other hand, PP2A may not be the targets of H9N2/G1 in the upstream of p38MAPK signaling pathways since H1N1 also induced PP2A activation in primary macrophages.

Despite the activation of PP2A, the levels of H9N2/G1-induced p38MAPK phosphorylation and the subsequent TNF-alpha production were still much higher than those in cells with H1N1 infection. This high level of p38MAPK phosphorylation raises a question on its roles in the virus infection. Previous study shows that p38MAPK facilitates influenza virus entry in human airway epithelial cells [25]. However, there was no significant difference in the levels of nucleoprotein in H9N2/G1-infected PBMac at 0, 4 and 8 h.p.i. with or without SB203580 treatment suggesting that p38MAPK does not facilitate the virus entry into primary human blood macrophages (Figure S1C). Whether this high level of p38MAPK phosphorylation contributes to other mechanisms that favor the virus replication needs to be further investigated.

The mechanisms of PP2A regulated cytokine production in influenza virus infections remain investigated. In this study, we used okadaic acid to inhibit the catalytic activity of PP2A. Since the substrate specificity of PP2A is determined by the combination of regulatory subunits and conserved catalytic subunits [26], the target of regulatory subunits of PP2A that are involved in suppressing TNF-alpha production will be our future study.

## 3. Experimental Section

### 3.1. Reagents and Antibodies

Specific antibodies against phosphorylated p38MAPK, ERK1/2 and JNK were purchased from Cell Signaling Technology (Beverly, MA, USA), while antibodies specific to MKP-1 and actin were purchased from Santa Cruz Biotechnology (Dallas, TX, USA). Antibodies specific to PP2A C subunit were purchased from Millipore. Specific inhibitors against p38MAPK (SB203580) and PP2A (okadaic acid) were purchased from Merck Millipore (Billerica, MA, USA).

### 3.2. Cells and Viruses

Human monocyte-derived macrophages from healthy donors (Hong Kong Red Cross Blood Transfusion Service) were prepared as previously described [10,12]. In brief, blood mononuclear cells were separated by Ficoll–Paque centrifugation and purified by the adherence method. Monocytes were differentiated in RPMI 1640 (Life Technologies, Carlsbad, CA, USA) supplemented with 5% heat-inactivated autologous plasma. Differentiated macrophages were obtained after culturing for 14 days. Influenza viruses including A/Quail/Hong Kong/G1/97 (H9N2/G1) and A/Hong Kong/54/98 (H1N1) were grown in Madin–Darby canine kidney cells and were purified by pre-adsorption to and elution from turkey red blood cells. Virus infectivity was determined by titration on Madin–Darby canine kidney cells.

### 3.3. Virus Infection

Macrophages were infected with the viruses at a multiplicity of infection of two for 30 min at 37 °C. The supernatant containing the virus inoculum was then removed, and the cells were incubated in macrophage serum-free medium (Life Technologies, Carlsbad, CA, USA) supplemented with 0.6 mg/mL penicillin and 60 mg/mL streptomycin. The mock-treated control was incubated with the buffer under parallel conditions.

### 3.4. Protein Extraction

Proteins were harvested by using SDS-lysis buffer containing 1% Triton X-100, 0.5% NP-40, 150 mM NaCl, 10 mM Tris–HCl (pH 7.4), 1 mM EDTA, 1 mM EGTA (pH 8.0), 1% SDS, 0.2 mg/mL PMSF, 1 μg/mL aprotinin, 1 mM sodium orthovanadate, 2 μg/mL pepstatin, 2 μg/mL leupeptin and 10 mM sodium fluoride.

### 3.5. Quantitative Reverse Transcription-PCR Analyses

Total RNA was extracted with TRIzol reagent (Life Technologies, Carlsbad, CA, USA) according to the manufacturer’s instructions. The cDNA was synthesized from total RNA with oligo (dT) primers and Superscript II reverse transcriptase (Life Technologies, Carlsbad, CA, USA. The levels of mRNA encoding TNF-alpha were assayed with TaqMan Gene Expression Assays (Life Technologies, Carlsbad, CA, USA).

### 3.6. Quantitative Analyses of TNF-Alpha by Enzyme-Linked Immunosorbent Assays (ELISA)

Supernatant samples of macrophage cultures were collected at the indicated time points after infection and irradiated with UV light before the level of TNF-alpha was measured with specific ELISA Kits (R&D Systems, Minneapolis, MN, USA).

### 3.7. Protein Phosphatase 2A Activity Assay

Cells were washed with pre-chilled PBS once and incubated with by phosphatase lysis buffer (20 mM HEPES, 10% Glycerol, 0.5% NP-40, 1 mM EGTA, 2 mM EDTA, 0.1 mM MgCl2, 20 mM Imidazole-HCl, 1 mM benzamidine, 10 μg/mL aprotinin, 10 μg/mL leupeptin, 1 mM PMSF, pH 7.0) on ice for 5 min. The cells were collected by scrapers, sonicated three times for 10 s on ice and then centrifuged at 2000× *g* for 5 min at 4 °C. The supernatant was collected and the protein concentration was measured by BCA kit (Thermo Scientific, Waltham, MA, USA). PP2A activity was carried out by malachite green-based PP2A Immunoprecipitation Phosphatase Assay Kit (Millipore, Billerica, MA, USA), following the manufacturer’s protocol.

### 3.8. Statistical Analysis

Data obtained from two groups with different treatments were analyzed by two-tailed, paired Student’s *t*-test (statistical significance if *p* < 0.05).

## 4. Conclusions

Taken together, our results show that H9N2/G1 induced a higher level of TNF-alpha production than that of H1N1 viruses and this hyperinduction was mediated by p38MAPK. PP2A involved the regulation of p38MAPK activation and TNF-alpha production in H9N2/G1 infection. However, H9N2/G1 did not induce the expression of PP2A indicating that the activation of PP2A is not mediated by p38MAPK in virus-infected PBMac. Our results may give new insights into the control of cytokine expression in influenza virus infection and may provide information in the development of therapeutics in controlling cytokine dysregulations in the disease process.

## Supplementary Information
