# Supplementary material for: A Role for Protein Phosphatase 2A in Regulating p38 Mitogen Activated Protein Kinase Activation and Tumor Necrosis Factor-Alpha Expression during Influenza Virus Infection
Source: Int J Mol Sci. 2013 Apr 2;14(4):7327–40. doi: 10.3390/ijms14047327 (PMC3645688; doi:10.3390/ijms14047327)
Supplement: Supplementary file 1 [file ijms-14-07327-s001.docx]

**Supplementary Information**

**Figure S1.** Effects of the relative specific p38MAPK inhibitor. (**A**) Primary human monocyte-derived macrophages (PBMac) were mock-treated or treated with 2.5 μM SB203580 for 30 min prior to H9N2/G1 virus infections. The phosphorylation levels of p38MAPK were examined at 2 h.p.i. by using Western blot analysis. Representative figure of experiments from five independent blood donors was shown. Actin was used as a loading control; (**B**) PBMac were treated with the indicated doses of SB203580 for 8, 12, 16 and 24 h. MTT assay was performed to measure the cell survival. The cell survival levels were calculated from at least three independent blood donors and were expressed in percentages ± SD, relative to the solvent control; (**C**) PBMac were treated with DMSO or 2.5 μM SB203580 for 30 min prior to H9N2/G1 infection. At 0, 4 and 8 h.p.i., total protein lysates were harvested. Levels of viral nucleoprotein (NP) were measured by Western blot analysis. Actin was used as a loading control. Band intensities were measured by Quantity One imaging software (Bio-Rad). The relative intensities of NP compared to actin were presented graphically in the lower panel. Representative figure of experiments from at least three independent blood donors is shown. MTT,3-(4,5-Dimethylthiazol-2-yl)-2,5-diphenyltetrazolium bromide; DMSO, dimethylsulphoxide; h.p.i., hour post infection; SD, standard deviation.

**
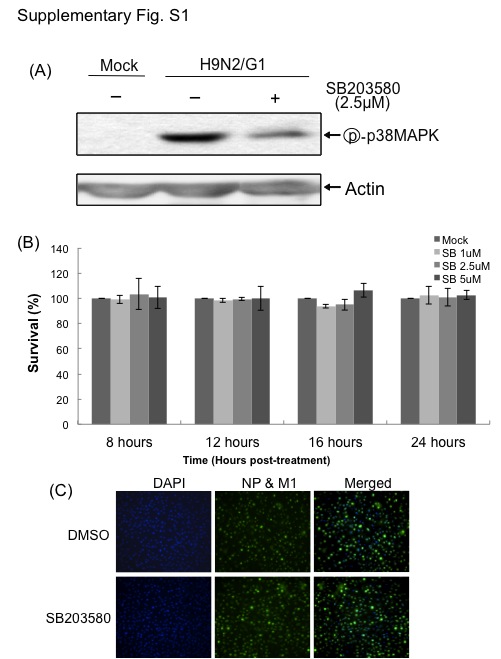
**

**
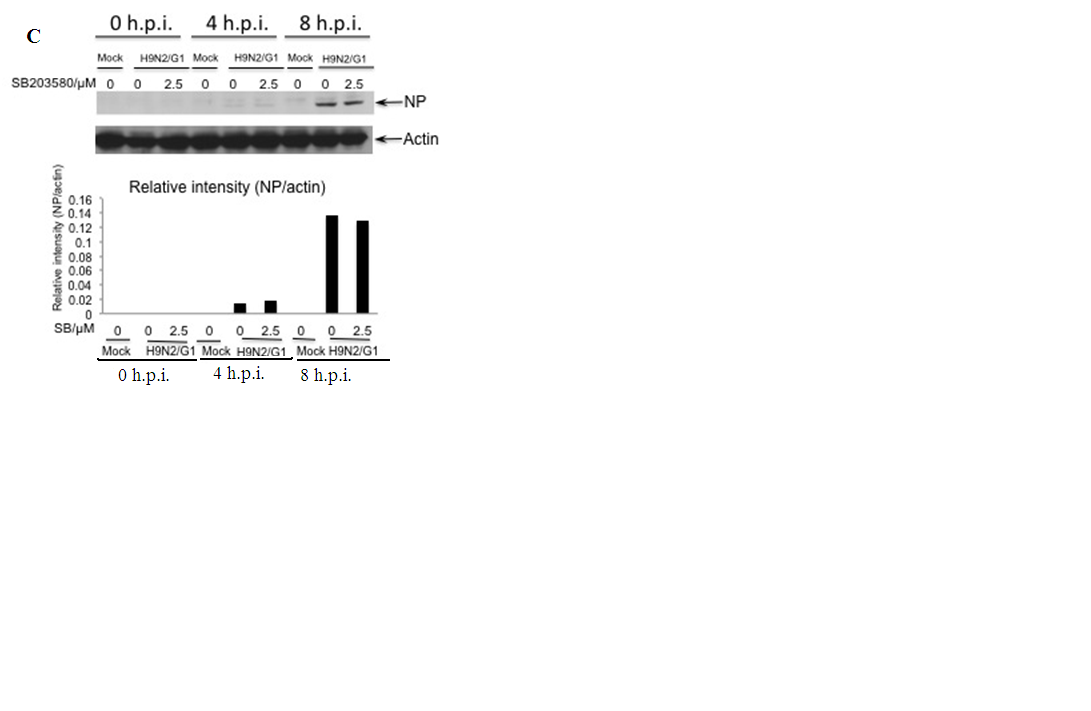
**

**Figure S2.** Viability of macrophages upon okadaic acid treatment. Primary human monocyte-derived macrophages were treated with 0, 15 or 25 nM okadaic acid (OA) at the indicated time points. The cell viabilities were measured by MTT assay. The survival rate of treated cells were calculated from four independent blood donors and were expressed in percentages ± SD, relative to DMSO control. MTT,3-(4,5-Dimethylthiazol-2-yl)-2,5-
diphenyltetrazolium bromide; DMSO, dimethylsulphoxide; h.p.i., hour post infection;
SD, standard deviation.


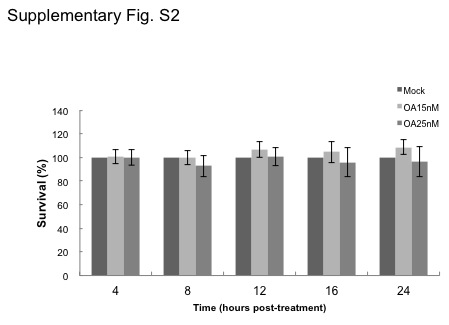


**Figure S3.** Effect of okadaic acid on p38 MAPK phosphorylation. Lane 1 and 2: Primary human monocyte-derived macrophages (PBMac) were mock-treated (0 nM) or treated with 50 nM okadaic acid. After 2.5 h incubation, cell lysates were collected and phosphorylation levels of p38MAPK were determined by Western blot analysis. Lane 3: PBMac were treated for 30 min and were then infected with H9N2/G1. Phosphorylation level of p38MAPK at
2 h.p.i. was determined by Western blot analysis as positive control.


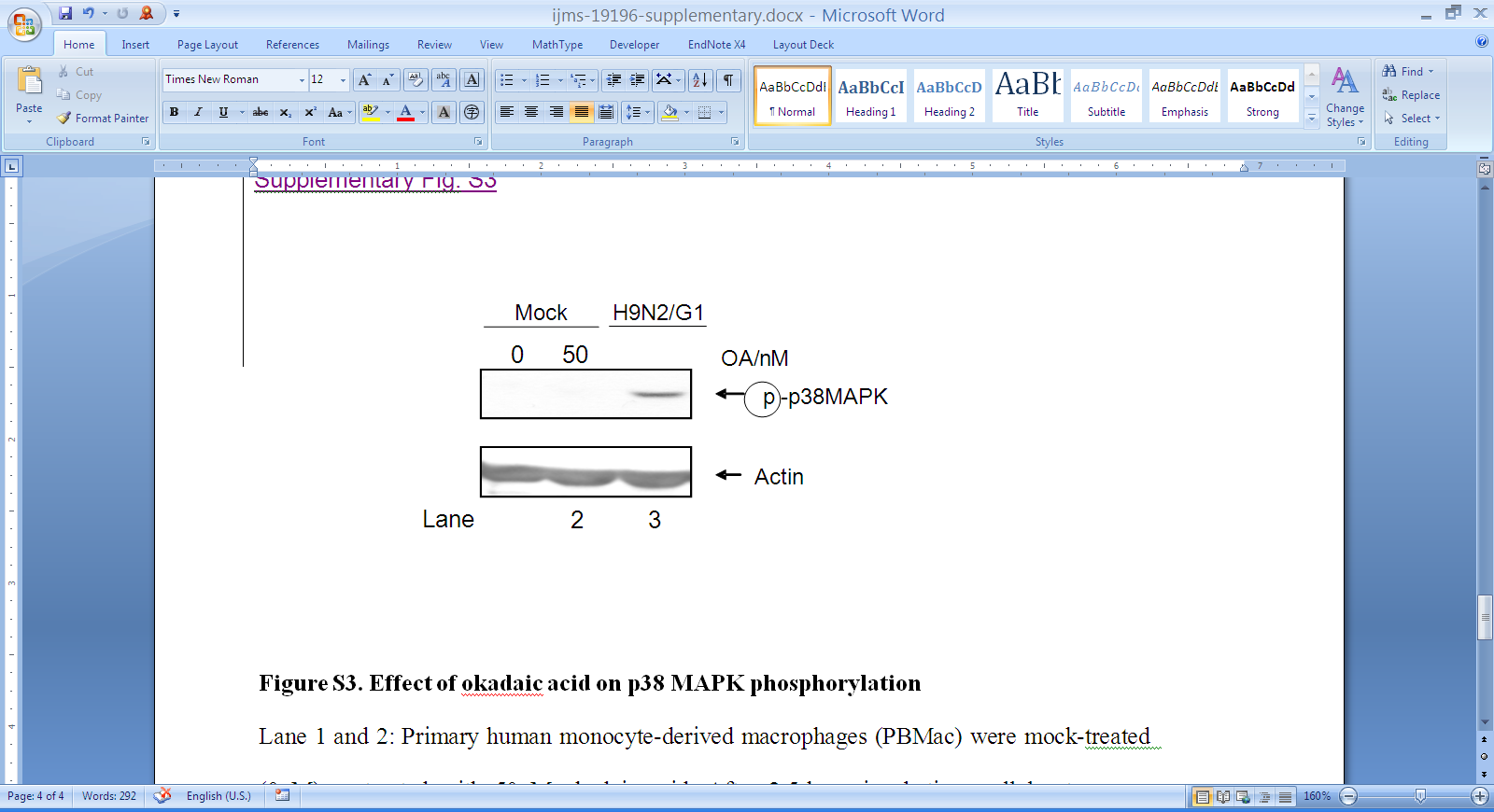


© 2013 by the authors; licensee MDPI, Basel, Switzerland. This article is an open access article distributed under the terms and conditions of the Creative Commons Attribution license (http://creativecommons.org/licenses/by/3.0/).
